# Investigations of silk fiber/calcium phosphate cement biocomposite for radial bone defect repair in rabbits

**DOI:** 10.1186/s13018-017-0529-8

**Published:** 2017-02-21

**Authors:** Lei Zhou, Chunjie Hu, Yingjun Chen, Shiqi Xia, Jinglong Yan

**Affiliations:** 1 Department of Orthopedics, The Second Affiliated Hospital of Harbin Medical University, Harbin, 150081 China; 2 Department of Obstetrics and Gynecology, The Fourth Affiliated Hospital of Harbin Medical University, Harbin, 150081 China; 3 Department of Orthopedics, The People’s Hospital of Shangqiu City, Henan, 476000 China

**Keywords:** Radius, Tissue engineering, Silk fiber, Calcium phosphate cement

## Abstract

**Background:**

This study aimed to investigate the effects of silk fiber (SF)/calcium phosphate cement (CPC) biocomposite on repairing radial bone defects in rabbits.

**Methods:**

Four-month-old New Zealand rabbits were selected to create a bilateral radial bone defect model and divided into four groups according to implanted material: SF/CPC, SF/CPC/particulate bone (PB), PB, and control (C). The specimens were removed at four and eight postoperative weeks for general observation, X-ray examination, tissue slicing, scanning electron microscopy (SEM), and biomechanical testing.

**Results:**

Postoperative X-ray showed no bone defect repair in group C and different degrees of bone defect repair in the other three groups. Imaging, histology, and SEM showed the following: group SF/CPC formed fine trabecular bone in week 4, while the maximum bending load in group SF/CPC in week 4 was significantly different from those in the other groups (*P* < 0.05).

**Conclusions:**

SF/CPC has good biocompatibility and bone-inducing ability, demonstrating its bone defect-repairing ability.

## Background

Trauma, infection, tumors, and other congenital diseases have often caused bone defects in recent years [1]. The purposes of repair and reconstruction are to restore bone structure and function as quickly and completely as possible [2]. Therefore, studies of bone tissue engineering are gradually increasing. Ideal bone defect repair materials should have the following qualities [3–5]: (1) sufficient mechanical properties and good biomechanical adaptation; (2) bone conduction; (3) osteoinductivity; (4) ability to provide osteoblasts; (5) ability to directly form bones; (6) good material–bone interface; and (7) good shapability. Currently, no clinically applied bone repair material can meet all of the above criteria [6]; however, in clinical application, a material often just needs to meet some conditions when used to repair a bone defect, so we can select suitable materials according to the specific repair circumstances. Several types of composite materials are discussed below; however, achieving ideal bone defect repair is the key in bone tissue engineering.

Autologous bone is the best material for repairing bone defect and can lead to ideal bone healing. However, autologous bone is difficult to sample and available in small quantities, and sampling will inevitably increase the patient’s pain and risk of infection during the treatment process [7]. Although allogeneic bone can be easily sampled, its sources are richer than those of autogenous bone and it has some excellent tissue characteristics like autologous bone, it normally causes immune rejection due to antigenic differences among different species; thus, it has such biosafety risks such as leading to infections and tumorigenesis. At the same time, sample preparation, handling, and storage costs are high, so its applications are greatly limited [8, 9]. Therefore, further research is needed to find materials that can both meet the mechanical requirements and achieve ideal bone fusion. Wu et al. achieved good results with one scaffolding material used in the clinical setting [10], but due to its slow degradation, large cystic characteristic, and insufficient flexibility, its applications have since been limited. Silk fiber (SF) has a long history of being used as a natural biological material in clinics [11], but since it is fine, its aggregation is not guaranteed and it exhibits ideal strength only when used in large amounts. One study [12] used the fiber-reinforcing principle to add SF to calcium phosphate cement (CPC) for in vitro experiments and confirmed that SF can enhance the mechanical strength of CPC. To further study its ability to repair bone defects, we performed in vivo animal experiments. SF can enhance the mechanical and biological properties of bone tissue engineering materials as well as accelerate ossification and repair bone defects.

## Methods

### Materials and instruments

A total of forty-eight 4-month-old New Zealand rabbits of either sex (provided by the Experimental Animal Center, The First Affiliated Hospital of Harbin Medical University, Harbin, China) with a body weight of 1.8–2.2 kg were used in the experiment. Mulberry silkworm silk, CPC (Shanghai Rebone Biomaterials Co., Ltd., Shanghai, China), anhydrous Na_2_CO_3_ (Tianjin Tanggu Chemical Reagent Factory, Tianjin, China), an Instron5569 universal mechanical testing machine (provided by the School of Materials Science and Engineering, Harbin Institute of Technology, Harbin, China), a Faxitron MX 20 Film X-ray machine (USA), a Thermo HM325 tissue slicer (provided by the Laboratory of Tissue Engineering, The Second Affiliated Hospital of Harbin Medical University, Harbin, China), and a 13 S-3400N scanning electron microscope (provided by the Laboratory of Electron microscopy, Harbin Medical University, Harbin, China) were also used. This study was carried out in strict accordance with the recommendations in the Guide for the Care and Use of Laboratory Animals of the National Institutes of Health. The animal use protocol has been reviewed and approved by the Institutional Animal Care and Use Committee (IACUC) of Harbin Medical University.

### Animal grouping and modeling

The 48 New Zealand rabbits were prepared for the bilateral radial bone defect model: 2 mL/kg chloral hydrate (10%, *m*/*m*) was injected via the ear vein for anesthesia, and then one 25-mm-long incision was made on the radial side of the bilateral forelimbs to isolate and expose the radius; one rongeur was then used to remove approximately 15 mm of periosteum-containing bone from the lower radial section. According to different implant materials for the bone defect repair, all of the models were divided into four groups (*n* = 12 each): SF/CPC (implanted SF/CPC biocomposite), SF/CPC/particulate bone (PB) (implanted SF/CPC biocomposite and tiny particulate bone), PB (simply implanted tiny particulate bone), and control (without implanting any materials). All of the rabbits received a postoperative plaster bandage for external fixation and were housed in the same environment; six rabbits in each group were killed in postoperative weeks 4 and 8 for general observation and imaging inspection of 12 bone defect sites (bilateral) at each designed time point, among which eight sites were also subjected to biomechanical testing and four were subjected to histology and scanning electron microscopy (SEM).

### Preparation of SF

Cleaned mulberry silks were put into 0.3% sodium carbonate solution at 100 °C for 30 min and then washed twice for 10 min using distilled water. Pure SF strands were cut to 3-mm lengths and conserved after X-ray irradiation sterilization.

### Preparation of PB

After satisfactory anesthesia, each laboratory animal was placed in the prone position, the skin in the iliac region was prepared and anesthetized, and paved the sterile tower. Approximately 1.5 cm × 1.5 cm of iliac bone was then cut off from one side, and after the soft tissue and periosteum on the surface were removed, this iliac bone was fully ground in saline using a drill to 5 mm in diameter; the bone particle mixture was then filtered using a 500-μm-diameter mesh, followed by centrifugation at 3000 r/rain for 5 min (effective centrifugation radius 10 cm); the supernatant was then removed to obtain bone particle sediment 500 ixm in diameter.

### Preparation of SF/CPC biocomposite

SF and CPC were uniformly mixed at a mass ratio of 1:20, to which an appropriate amount of curing liquid was added to prepare a biocomposite approximately 4 mm in diameter and 15 mm in length. After being sterilized with ethylene oxide in a fumigation box, the biocomposite was stored for future use. The same method was used to mix SF, CPC, and tiny PB with mass ratios of 1:20:20 to prepare a composite material of the same length.

### Main observation indexes

#### General observation

The postoperative general conditions, activities, peripheral blood supply, and wound infections of the animals were observed; meanwhile, gentamicin was routinely subcutaneously injected to prevent infection. All of the animals were killed 4 and 8 weeks postoperative, and the bilateral ulnoradial specimens were removed for visual observation of the bone defect repair.

#### Imaging

The specimens obtained in weeks 4 and 8 (*n* = 12) were subjected to X-ray for observation of the formation conditions of new bone or bone callus at the biocomposite implantation site. The X-ray exposure conditions were 45 kV, 100 mA, and 0.12 s.

#### Histological observation

The specimens were obtained during weeks 4 and 8 and included ±5 mm of the normal bone above and below the biocomposite implantation site. After removal of the surrounding periosteum and soft tissue, the specimens were placed in a 4% paraformaldehyde solution (*m*/*m*) for 24-h fixation and then soaked in a 10% ethylenediaminetetraacetic acid solution (*m*/*m*) for 4-week decalcification. After thorough removal of the Ca content, the specimens were subjected to dehydration, hyalinization, paraffin embedding, slicing, dewaxing, and water rinsing followed by hematoxylin and eosin staining; the formation of trabecular bone as well as the growth of bone cells inside the biocomposite were then observed under optical microscopy.

#### Scanning electron microscopy

The specimens obtained during weeks 4 and 8 (*n* = 12) were fixed in 3% glutaraldehyde solution (*m*/*m*), dried, and gold-sputtered, followed by SEM observation of the biocomposite–bone stem interface as well as the ultrastructure of the newly formed bone.

#### Biomechanical testing

The fresh radial bone specimens of the three groups obtained during weeks 4 and 8 (*n* = 8) were processed by first removing the surrounding soft tissue and periosteum and then retaining the bone ends 1 cm above and below the bone defect for a three-point bending test (loading span 10 mm; fulcrum span 16 mm; the specimen was placed horizontally on the mechanical testing machine for the detection; the maximum bending load was set as the measure index to indicate its anti-bending strength) using one electronic universal testing machine, and the data were recorded for the following statistical analysis.

#### Statistical analysis

SPSS 18.0 statistical software was used, and the maximum bending load is expressed as $\overline{x}$ ± s. One-way analysis of variance was used to compare the maximum bending load among groups, while the SNK-q test was used to make pairwise comparisons among the groups. Values of *P* < 0.05 were considered statistically significant.

## Results

### General observation

The visual observation of bilateral radial bone in weeks 4 and 8 revealed that group C had no bone defect healing at the two time points, whereas the other three groups exhibited different degrees of bone defect healing. The defects were detected using SEM, biomechanical measurements, histological observations, and radiographic observations. In week 4, the SF/CPC/PB group showed better biocomposite–bone combination and new bone formation than the SF/CPC group, while the latter showed a less smooth surface; in week 8, the SF/CPC, SF/CPC/PB, and PB groups all showed better bone defect healing as well as complete new bone formation on the bone surface.

### Imaging

**Fig. 1:**
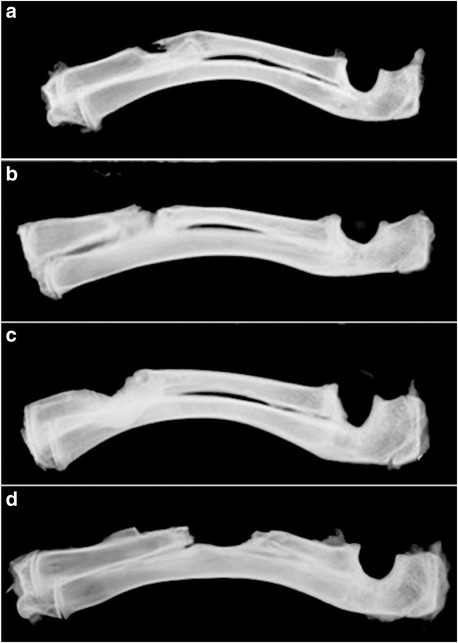
In week 4, X-ray: group SF/CPC exhibits non-complete bone defect repair, the bone cortex is discontinuous, and the interface line of bone ends is visible (**a**); group SF/CPC/PB exhibits the completeness of filling material at the bone defect site, new bones are rich, and the bone cortex is fine and continuous (**b**); group PB exhibits not-yet-repaired bone defect, but the trabecular bone tends to neat arrangement, and the bone defect exhibits bone connection (**c**); and group C exhibits low-density shadow of soft tissue at the bone defect site, and the bone defect is not repaired (**d**)

**Fig. 2:**
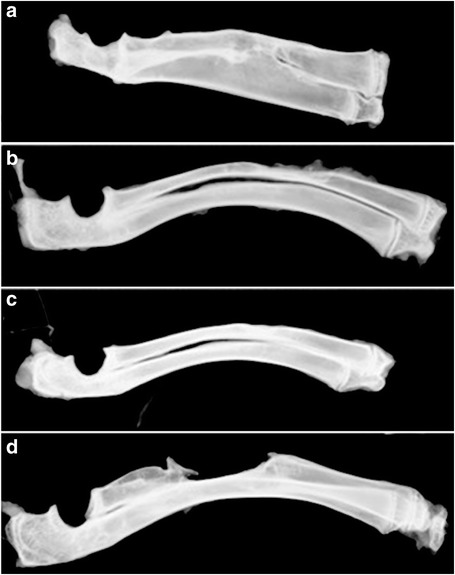
In week 8, X-ray: group SF/CPC exhibits more complete bone cortex, the interface line between the bone and implant material disappears, the trabecular bone tends to neat arrangement, the medullary cavity is recanalized, and the shape is good (**a**); group SF/CPC/PB exhibits complete bone defect repair, the fusion of the bone ends is complete, the bone cortex is continuous, the medullary cavity is recanalized, and the shape is good (**b**); group PB exhibits complete bone defect repair, the bone cortex is continuous, the medullary cavity is recanalized, and the shape is good (**c**); and group C exhibits no bone defect repair, and the ends at the bone defect site develop into bone sclerosis (**d**)

Group C showed no bone repair at the two postoperative time points and hardened bilateral bone ends on X-ray imaging. The other three groups exhibited different degrees of bone repair: the implanted material gradually combined with the backbone, and the bone fracture line gradually disappeared. In week 4, the SF/CPC group exhibited more trabecular bone disorders, less of an unobstructed medullary cavity, and poorer cortical bone continuity than the SF/CPC/PB group (Fig. 1); in week 8, the boundary lines between the implanted material and the backbone in the three groups disappeared, the materials and the bone fully integrated, the trabecular bone tended to be neat, the cortical bone was continuous, and the medullary cavity was recanalized (Fig. 2).

### Histological observation

**Fig. 3:**
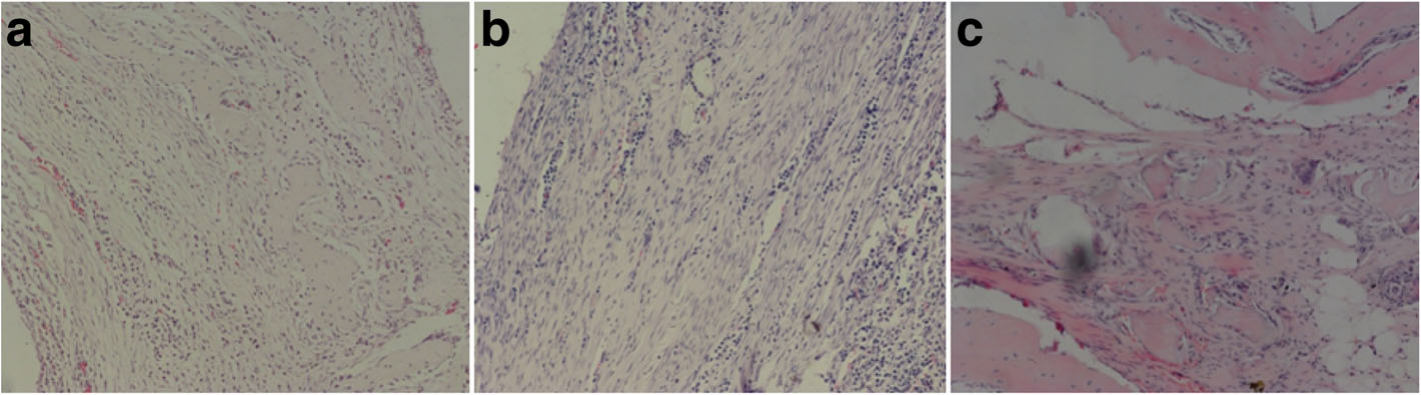
In week 4, histological examination shows group SF/CPC (**a**) and SF/CPC/PB (**b**) have the growth of a large number of bone cells, but the trabecular bone in group SF/CPC is smaller than group SF/CPC/PB and PB (**c**), and the structure is blurred (HE staining ×100)

**Fig. 4:**
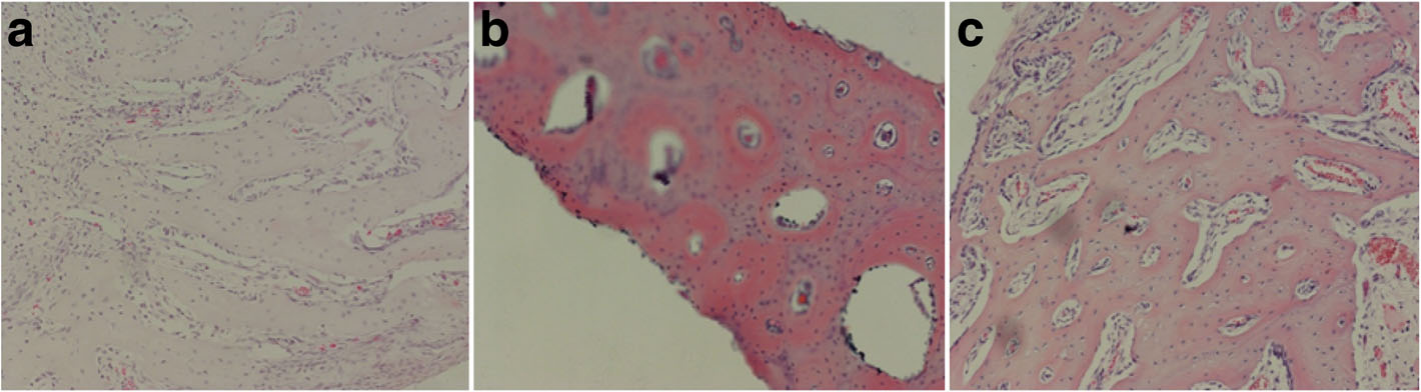
In week 8, histological examination shows group SF/CPC (**a**), SF/CPC/PB (**b**), and PB (**c**) show significantly increased bone lacuna, the trabecular bone becomes coarse and mature and has clear structure; meanwhile, the blood sinusoid forms clearly (HE staining ×100)

In week 4, the SF/CPC/PB and PB groups exhibited the ingrowth of a large group of bone cells in the fully formed trabecular bone and the SF/CPC group exhibited the formation of a small amount of trabecular bone (Fig. 3); in week 8, the three groups all exhibited the growth of a large number of bone cells, while the trabecular bone was more complete and mature and had already replaced the interface line (Fig. 4).

### Scanning electron microscopy

**Fig. 5:**
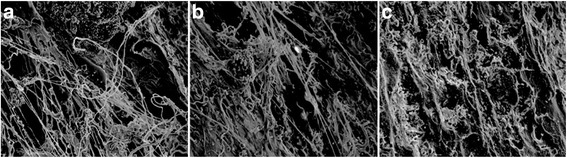
In week 4, SEM shows that group SF/CPC (**a** ×450) and SF/CPC/PB (**b** ×700) shows the dressing and deposition of bone along SF. SF appears as an irregular shape, the bone deposition in group SF/CPC is less and incomplete than those in group SF/CPC/PB and PB (**c** ×500), and no complete trabecular structure can be seen

**Fig. 6:**
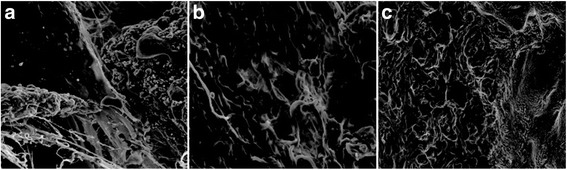
In week 8, SEM shows increased bone deposition in all the groups than those in week 4, SF in group SF/CPC (**a** ×800) and SF/CPC/PB (**b** ×1000) are completely wrapped, the trabecular structure is clear, and the shapes are more complete; group PB (**c** ×90) shows good connection between the bone defect and bone ends, and the trabecular structure is clear and complete

In week 4, the SF/CPC and SF/CPC/PB groups all exhibited the dressing and deposition of bone along the SF, while the PB group exhibited more bone deposition within the trabecular bone and the bone deposition in the SF/CPC group was less than those in the SF/CPC/PB and PB groups; the trabecular bones in the three groups exhibited disorganization at the interface (Fig. 5). In week 8, the SF/CPC group exhibited gradually increased bone deposition, while the SF/CPC/PB and PB groups showed no significant change; the trabecular bone tended to be neatly arranged, and SF degradation was visible. The bone deposition amounts in the three groups gradually increased with time, the trabecular bone gradually became remodeled, and the implanted material–bone end combination became firmer (Fig. 6).

### Biomechanical testing

The three-point bending test performed in weeks 4 and 8 showed that, in week 4, the maximum bending load in the SF/CPC group was significantly different from those of the SF/CPC/PB and PB groups (*P* < 0.05, Table 1), but the difference between SF/CPC/PB and PB was not statistically significant (*P* > 0.05, Table 1). In week 8, the pairwise comparison of maximum bending load among the three groups showed no statistical significance (*P* > 0.05, Table 1). Table 1 shows a comparison of maximum bending load among the three groups in weeks 4 and 8 (*N*, mutual ± s, *n* = 8).

**Table 1:** Comparison of maximum bending load among the three groups in weeks 4 and 8 (*N*, $\overline{x}$ ± s, *n* = 8)

Group	Week 4	Week 8
SF/CPC	71.99 ± 10.25	101.71 ± 12.63
SF/CPC/PB	87.37 ± 6.94*	103.52 ± 17.09
PB	91.13 ± 6.17	104.69 + 12.82
*F*	12.480	0.180
*P*	0.002	0.914

*SF/CPC* silk fiber/calcium phosphate cement

*Compared with group SF/CPC, *P* < 0.05

## Discussion

Our group previously found that calcium polyphosphate fibers can improve the mechanical properties of tissue-engineered bone and provide good bore diameter and porosity after degradation to facilitate bone ingrowth and repair based on studies of calcium polyphosphate fibers, calcium phosphate bone cement, and small-particle bone repair bone defect. Based on these relevant studies, we used SF as reinforcing materials to repair the bone defect due to its good biological properties [4, 13, 14]. SF is a fibrous protein that is produced mainly by silkworms and spiders. Its unique mechanical properties, including a tunable biodegradation rate and the ability to support the differentiation of mesenchymal stem cells along the osteogenic lineage, have made SF a favorable scaffolding material for bone tissue engineering. SF can be processed into various scaffold forms, combined synergistically with other biomaterials to form composites and chemically modified components, which provides an impressive toolbox and allows SF scaffolds to be tailored to specific applications.

This study was based on the fiber-reinforcing principle in which we prepared SF into short fibers and then combined it with CPC, so the composite had both enhanced rigid strength and increased porosity; furthermore, with the constant degradation of SF in vivo, the porosity of the biocomposite was further increased, allowing it to promote bone cell ingrowth, promote Ca deposition, and accelerate bone replacement, thus facilitating the gradual absorbance of bone cement and achieving the purpose of ideal bone defect repair [15]. At the same time, the addition of SF can reduce the occurrence of CPC loosening after implantation, reduce the risk of pulmonary embolism [16, 17], and increase the safety of CPC.

The use of SF/CPC biocomposite in bone tissue engineering has theoretical and practical feasibility. The tissue slices in this study showed that, due to the addition of the small bone particles, the SF/CPC/PB and PB groups exhibited bone cell growth and trabecular bone formation in the early experimental stage, and over time, the trabecular structure tended to be complete and robust, indicating that bone particles play an important role in the bone creeping and substitution process [18]. The SF/CPC group exhibited only slight trabecular bone formation in week 4, which may have been due to the following: (1) bone replacement and bone formation start from the interface and gradually developing toward the internal portion of the composite and (2) since the SF/CPC biocomposite lacks bone mesenchymal stem cells (BMSCs) and cell growth factors, BMSCs gradually migrate into the biocomposite and then release cell growth factors [19, 20]. In week 8, histological observation revealed the formation of a large amount of trabecular bone, indicating that the SF/CPC biocomposite may meet the bone formation conditions: (1) when SF degrades in vivo, the porosity of the composite increases; (2) with gradual BMSC growth and proliferation, they can continue to release cell growth factors; and (3) SF itself can induce vascularization, thus increasing the local nutrient supply. The biomechanical testing results revealed that, in week 4, the mechanical strength in the SF/CPC group was lower than those of the SF/CPC/PB and PB groups, which may be caused by the structure of trabecular bone [21, 22]. Since SF will aggregate when it meets water, it will result in non-uniform distribution of the implant material; during the bone induction process, the growth speed of bone cells and the calcification extents of minerals differ; the SF/CPC/PB and PB groups exhibited a large range of bone formation in the early implantation stage, but the trabecular bone in the SF/CPC group formed earlier and was thinner, thus making it impossible to achieve the desired mechanical strength. In week 8, the maximum bending loads among the three groups showed no significant difference (*P* > 0.05), indicating that the SF/CPC group also formed a strong trabecular structure at this time. During the trabecular bone formation process, SF constantly biodegrades, thus producing a variety of amino acids; together with the vasoactive roles of SF, it is conducive to bone cell growth. Meanwhile, CPC is also gradually degraded, resulting in an extracellular environment rich in Ca^2+^ and P^3+^, thus helping increase the Ca deposition among the pores. This study prepared a new SF/CPC biocomposite and verified the biocompatibility and osteoinductivity of SF in animal experiments. The definition of bone defect repair is not simply fusion, it also includes whether the post-fusion mechanical strength is similar to that of normal tissues (including flexural strength). The biomechanical testing results in week 8 showed that the new SF/CPC biocomposite exhibited strength similar to that of the fine bone particles; therefore, applying SF/CPC in bone tissue engineering can reduce the requirement of autologous bone particles to a certain extent. SF/CPC biocomposites are currently at the basic experimental stage; although there have been some achievements, it is not yet widely used in the human body. Many problems remain to be solved: SF is biodegradable, but how is its degradation time connected with the speed of osteogenesis and when SF is biodegraded more quickly than the bone is formed, how can we ensure the mechanical strength of the new material? This study mixed SF and CPC at a ratio of 1:20 and achieved the desired results, but the best mixing ratio of SF and CPC remains unclear; thus, we have yet to determine how we can improve the porosity of the composite material to improve the early-stage osteogenesis speed and strength. SF aggregates easily and affects osteogenesis, so we must still determine how to improve the experimental methods to reduce the above phenomenon and achieve more uniform overall bone formation.

## Conclusions

In conclusion, our results and those of others suggest that novel composite self-hardening CPC scaffolds with fibers have high strength and other favorable characteristics such as the ability to promote osteogenesis, which may make this material useful in bone and other orthopedic repairs. The method of using absorbable fibers in grafts to provide strength and subsequently form macropores for bone growth may have wide applicability in other tissue engineering materials.

## Abbreviations

CPC: Calcium phosphate cement
PB: Particulate bone
SEM: Scanning electron microscopy
SF: Silk fiber
